# QuickBind: A Light-Weight And Interpretable Molecular Docking Model

**Published:** 2024-10-21

**Authors:** Wojtek Treyde, Nazim Bouatta, Seohyun Chris Kim, Mohammed AlQuraishi

**Affiliations:** Department of Systems Biology, Columbia University, New York, NY; Department of Systems Biology, Harvard Medical School, Boston, MA; Department of Systems Biology, Columbia University, New York, NY; Department of Systems Biology, Columbia University, New York, NY

## Abstract

Predicting a ligand’s bound pose to a target protein is a key component of early-stage computational drug discovery. Recent developments in machine learning methods have focused on improving pose quality at the cost of model runtime. For high-throughput virtual screening applications, this exposes a capability gap that can be filled by moderately accurate but fast pose prediction. To this end, we developed QuickBind, a light-weight pose prediction algorithm. We assess QuickBind on widely used benchmarks and find that it provides an attractive trade-off between model accuracy and runtime. To facilitate virtual screening applications, we augment QuickBind with a binding affinity module and demonstrate its capabilities for multiple clinically-relevant drug targets. Finally, we investigate the mechanistic basis by which QuickBind makes predictions and find that it has learned key physicochemical properties of molecular docking, providing new insights into how machine learning models generate protein-ligand poses. By virtue of its simplicity, QuickBind can serve as both an effective virtual screening tool and a minimal test bed for exploring new model architectures and innovations. Model code and weights are available at this GitHub repository.

## 1 Introduction

Small organic molecules (“ligands”) are a major class of drugs that act by binding protein targets, thereby affecting their functionality and interfering with the molecular pathways of diseases. Their distinct advantages, including ease of synthesis, administration, and cell permeability, render them indispensable as a pharmaceutical modality. In early-stage drug discovery, structure determination of protein-ligand complexes is a critical scientific tool, as it can provide an understanding of the molecular determinants of binding and enable further optimization of drug affinity and selectivity. It is however bottlenecked by costly and cumbersome experimental procedures, which computational “molecular docking” promises to overcome. When supplemented by an estimate of the strength of the binding interaction, computational tools can be used to virtually screen very large spaces of drug-like molecules [1] for viable drug candidates that can serve as starting hypotheses for subsequent experimental investigation and development [2, 3]. Existing computational methods achieve high-quality predictions at the cost of increasingly long runtimes, caused, in the case of conventional physical methods, by the need to sample numerous binding locations and poses, and in the case of machine learning (ML)-based methods by the complexity of the underlying neural computations that implicitly do the same.

Modern methods can be largely divided into molecular docking and co-folding. In molecular docking, an approximate protein structure is assumed to be known, whereas in co-folding both protein and ligand structures are predicted from scratch. Although a new development, co-folding has become the focus of much recent research activity, including ROSETTAFOLD ALL-ATOM (RFAA) [4], NeuralPlexer [5], UMOL [6], and ALPHAFOLD 3 (AF3) [7]. Nonetheless, for drug discovery campaigns against a known and potentially well-studied protein target, it is often unnecessary to predict protein structures independently for every ligand, making molecular docking an attractive alternative given its higher speed (due to the assumed rigidity of the protein).

ML-based docking methods can be further divided into targeted docking, which requires specifying the approximate binding pocket, and blind docking, which does not. The first ML method to tackle the latter is EquiBind [8], which predicts the isolated, bound conformation of the ligand and uses a keypoint alignment mechanism to identify the rotation and translation needed to dock the ligand into the binding pocket. Subsequent methods employ more complex architectures. In particular, TANKBind [9] and E3Bind [10] first partition the protein into functional blocks using P2Rank [11], predict the interaction of the given ligand with each block, and choose the final pose based on the predicted binding affinity (TANKBind) or confidence score (E3Bind). Both architectures include components inspired by ALPHAFOLD 2’s (AF2’s) Evoformer module [12]. Importantly, TANKBind predicts an intermolecular distance map that is converted into final coordinates by numerical post-optimization, whereas E3Bind operates on the ligand coordinates directly. FABind [13] builds upon E3Bind by integrating the prediction of the location of the binding pocket into the main model, such that the whole process becomes end-to-end differentiable. Another leap forward was made by DiffDock [14], a diffusion-based generative model that, starting from an input conformer, predicts changes in torsion angles as well as the transformation needed to dock the ligand into the protein. The inductive bias to focus only on relevant degrees of freedom coupled with a generative formulation led to large improvements in accuracy (and increased runtimes). More recently, advances have come from integration of protein language models [15], pretraining techniques geared towards molecular docking tasks, substantial increases in model size, and a shift towards generative approaches [14, 5, 7]. In combination, these trends have led to considerable increases in the computational cost of ML-based molecular docking, at levels prohibitive for virtual screening.

**Figure 1:**
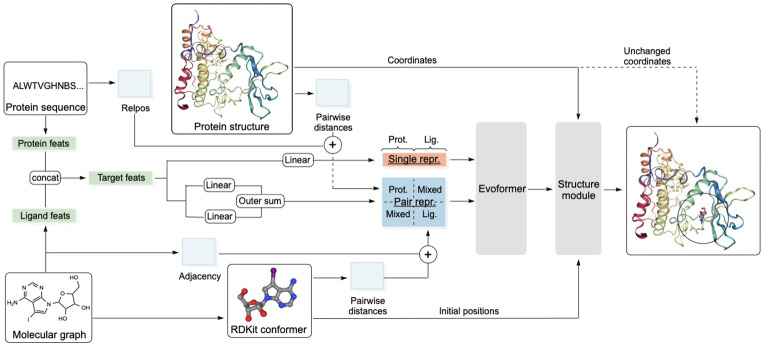
QuickBind architecture. A “single” representation is first constructed by concatenating embedded protein and ligand input features. A “pair” representation is then constructed from linear embeddings of the single representation, pairwise distances (of protein residues and ligands atoms, independently), relative positional encodings of protein residues, and the adjacency matrix of ligand atoms. The pair representation contains a protein and a ligand block, as well as mixed off-diagonal elements. The single and the pair representations are passed through a modified Evoformer stack, before the Structure module uses the updated single and pair representations as well as initial coordinates from an RDKit conformer [16] and protein coordinates from the input protein structure to dock the ligand into the binding pocket.

In this work we develop QuickBind, a light-weight method for rigid, blind molecular docking aimed at virtual screening applications by trading accuracy for speed. QuickBind performs well on the PDBBind test set, in particular when only unseen proteins are considered, and is substantially faster than DiffDock. Leveraging the AF2 architecture and problem formulation (Figure 1), QuickBind reasons over proteins using a residue-level representation in lieu of an atomistic one, permitting fast but implicit accounting of side chain flexibility. To accommodate additional degrees of freedom introduced by small molecule ligands, QuickBind incorporates a new framing strategy for the Invariant Point Attention (IPA) module of AF2. We also include a binding affinity prediction module to facilitate virtual screen applications, a capability typically absent from docking and co-folding methods. We showcase the utility and versatility of QuickBind by predicting binding affinities and structures for multiple important drug targets across various protein families, and investigate the interpretability of our model to better understand the biophysical basis of its predictions.

## 2 Methods

### 2.1 Dataset and evaluation metrics

We train and test QuickBind using the PDBBind [17] dataset and additionally assess it using the PoseBusters (PB) Benchmark [18]. PDBBind has been widely used to assess molecular docking methods using a temporal split proposed by EquiBind [8]. It contains crystal structures and binding affinities for ∼20,000 protein-ligand complexes; the training and validation sets comprise 16,379 and 968 complexes, respectively, both published before 2019, while the test set contains 363 complexes published in 2019 or later. There is no ligand overlap between the three partitions. PB contains 428 diverse complexes of unique proteins and drug-like ligands released since 2021, and is therefore disjoint from complexes in the PDBBind training set.

We quantify success based on the percentage of predictions with a symmetry-corrected ligand heavy atom root-mean-squared deviation (RMSD) of less than 2Å (“success rate”; [19, 20, 21, 22]). This criterion is widely used, as the residual deviation from the true bound conformation should not materially impact downstream analyses and optimizations. Evaluating models purely on success rate does not account for chemical and physical validity, but we assess these criteria using the PB suite [18]. Predictions that pass all PB tests and whose RMSD is below 2Å are deemed PB-valid.

### 2.2 Model architecture

QuickBind adapts the ALPHAFOLD 2 [12] architecture to the task of protein-ligand pose prediction (Figure 1). It takes as input protein sequence and structure as well as the chemical graph of the ligand and its 3D conformer (generated by the RDKit [16]). Inputs are combined to yield a unified first-order (“single”) representation and a second-order pairwise (“pair”) representation. These are then passed to a modified Evoformer stack that omits column-wise self-attention as multiple sequence alignments are not used. After processing by the Evoformer, a Structure module takes the updated single and pair representations as input as well as residue and ligand reference frames (using a new framing strategy for ligand atoms described below) and iteratively updates the ligand heavy atom coordinates. The Structure module is modified from AF2 so that the IPA module is gated [23], cross attention is performed between the single representations of the ligand and protein during the coordinate update step (Algorithm 3), and protein atoms are held fixed. QuickBind uses 12 Evoformer and 8 Structure module blocks. Full algorithm details are given in section SI.1.

AF2 uses reference frames to represent the geometry of protein residues: a translation vector corresponds to the coordinates of the C_*α*_ atom while a rotation matrix, anchored at these coordinates, is canonically constructed from the N, C_*α*_, and C coordinates to encode the orientation of the residue backbone. This representation is natural for linear or branched polymers but for atoms of arbitrary small molecules, there are no canonical frame construction approaches. With the emergence of co-folding methods, two recent models have proposed framing strategies for small molecules [4, 5], and QuickBind employs its own new approach. First, atom indices are reordered based on the canonical atom ranking of the RDKit [16]. For each heavy atom, its coordinates are treated as the C_*α*_ atom and the coordinates of its two adjacent atoms with the lowest indices are treated as the N and C atoms. If an atom has only one bond, we use a dummy atom (Algorithm 4). We then construct atom references frames using the same procedure employed for residue frames.

Using the above framing strategy, we found that during the IPA component of the Structure module, updating the rotation matrices of ligand atom frames improves performance, unlike the approaches of NeuralPlexer and RFAA which only update the translation component and passively reconstruct the frames. We note that while AF3 does not use reference frames to reason over protein-ligand complexes, it does use a framing strategy to calculate the predicted aligned error. AF3’s reference frames are constructed using the two closest atoms to a given center atom, and when an atom does not have two neighbors, the frame is ignored. We experimented with an analogous strategy but found that our approach works better.

For the loss function we adopt a modified version of the frame-aligned point error (FAPE) used by AF2. FAPE is computed by performing a set of alignments such that the predicted reference frame of each residue is aligned with its original reference frame in the target structure. FAPE is then the average, clamped RMSD between the predicted and target structures over all alignments. For molecular docking, FAPE can be reformulated as a combination of two components: the RMSD between predicted and target ligand atom positions based on ligand frame alignments and residue frame alignments. The final QuickBind model was trained using this combined FAPE loss, intermediate FAPE losses acting on the outputs of every Structure module block, and a Kabsch RMSD loss corresponding to the ligand RMSD after superimposition of the predicted and target ligand using the Kabsch algorithm [24].

Further model details are given in sections SI.2-SI.5. Before training the final QuickBind model, aspects of the architecture were optimized using two smaller variants, QuickBind-S and QuickBind-M (section SI.4). The results of this hyperparamater screen are summarized in Table SI.1.

## 3 Results

### 3.1 Model performance

**Figure 2:**
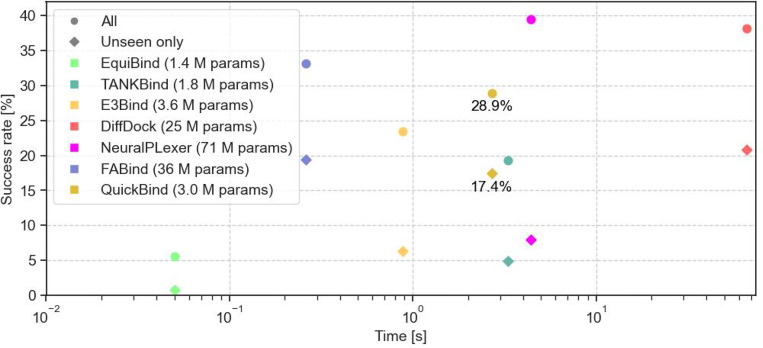
Success rates vs. average runtimes (summed over all complexes) for ML-based rigid docking methods on the PDBBind test set. Success rates are reported separately for all 363 complexes (circles) and for 144 complexes whose proteins are absent from the training and validation sets (diamonds). TANKBind was only evaluated on a subset of 142 unseen proteins by its original authors. Success rates are taken from original publications, except for NeuralPlexer’s success rate on unseen proteins as it was not originally reported. Runtimes do not include preprocessing and were determined on NVIDIA A40 GPUs using scripts provided in each method’s respective repository, without batching. NeuralPlexer runtime excludes acquisition of auxiliary inputs (*e.g.,* AF2 predictions) while TANKBind and E3Bind runtimes do not include P2Rank segmentation. E3Bind’s runtime was taken from its original publication since authors did not release the model weights and inference code.

We first assess QuickBind on the PDBBind test set (Figure 2) and compare it to other ML-based rigid docking methods including EquiBind, TANKBind, E3Bind, FABind, DiffDock, and NeuralPlexer (employed for blind molecular docking rather than co-folding). We exclude co-folding methods from this comparison as they have not been evaluated on PDBBind. For virtual screening—which can involve billions of molecules [25]—docking runtimes are a critical consideration. With an average runtime of 2.7s, QuickBind is orders of magnitude faster than traditional docking methods [8], DiffDock, and recent co-folding methods [4, 6, 7]. Accuracy-wise, QuickBind is middle-of-the-pack, outperforming most ML-based rigid docking methods except for FABind, DiffDock, and NeuralPlexer, particularly when generalizing to proteins excluded from the training set. Excepting FABind, these methods are all considerably slower, and FABind still uses an order of magnitude more parameters.

**Figure 3:**
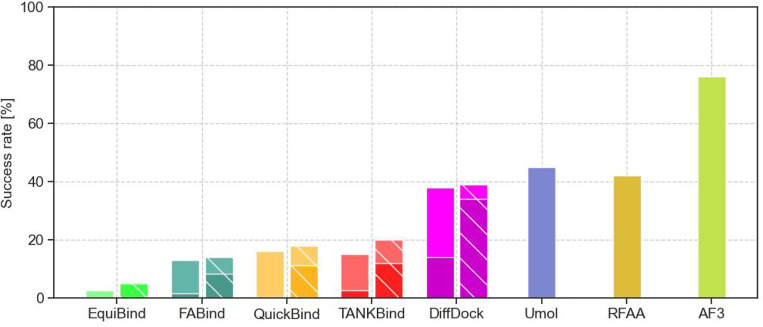
Success rates of ML-based rigid docking and co-folding models on the PB Benchmark, sorted in ascending order by model runtime (left-to-right). Target 7M31 was omitted for QuickBind because it is longer than 2,000 residues. Lighter colors correspond to all predictions while darker colors correspond to PB-valid predictions (docking methods only) and hatched bars to results after energy minimization. Success rates are taken from original publications [4, 6, 7, 18], except for FABind’s success rate as it was not originally reported. We found the EquiBind success rates to be 0.9% (all predictions), 0.2% (PB-valid predictions), 4.2% (all predictions after energy minimization), and 3.5% (PB-valid predictions after energy minimization), in rough agreement with the originally reported values (2.6%, 0.0%, 5.5%, and 4.8%, respectively).

Next, we assess QuickBind on the more challenging PB Benchmark (Figure 3) and include in our comparisons recent co-folding methods. QuickBind generally outperforms rigid docking methods (except DiffDock but including FABind) but trails co-folding methods. However, the speed gap between QuickBind and co-folding methods (which take minutes for a single prediction) is even more considerable, making QuickBind a compelling compromise between speed and accuracy. Figure SI.1 shows examples of highly accurate QuickBind predictions.

QuickBind forgoes a post-processing step common to most ML-based methods that enhance the chemical validity of predictions. As a result, QuickBind’s raw predictions do not generally pass the PB chemical and physical plausibility tests, largely due to incorrect bond lengths and angles (Figure SI.2, see also Figure SI.1a). As was noted in [18] however, such failures can typically be addressed by running a force field-based energy minimization post-prediction. Doing so does substantially improve the physical validity of QuickBind’s predictions and even slightly improves its success rate (Figure 3).

Given its speed, a natural use case for QuickBind is binding affinity prediction for virtual screening applications. To test this potential, we trained a simple neural network to use QuickBind’s single representations (Algorithm 5) to predict protein-ligand affinities. We used the same PDBBind split as for training QuickBind and PDBBind’s own affinity data. The resulting model is competitive with other affinity predictors (Table SI.3) despite not having been optimized architecturally or through hyperparameter tuning. This indicates that QuickBind has immediate utility for virtual screening and may be further improved using more advanced top models for affinity prediction.

### 3.2 Model interpretability

Having trained QuickBind, we set out to investigate whether it had learned ligand characteristics relevant for molecular docking. Beyond binding affinity, molecular features such as lipophilicity and cell permeability can influence the outcome of drug discovery programs. These features are strongly linked to the physicochemical properties of ligands, such as hydrophobic surface area and molecular weight, to the extent that expert rules for drug design often explicitly restrict drug-like molecules based on these properties. A model that implicitly encodes them may thus hold promise for drug design beyond accurate pose prediction.

**Figure 4:**
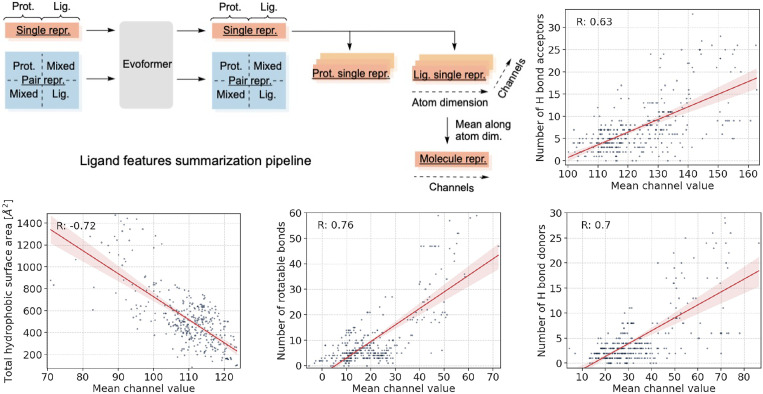
Interpretable physicochemical properties in QuickBind’s ligand representation. Processing the Evoformer’s single representation into separate protein and ligand representations followed by averaging the ligand’s atom dimension yields interpretable descriptors that correlate with channel values, including number of H-bond acceptors and donors, total hydrophobic surface area, and number of rotatable bonds.

To this end, we extracted the single ligand representation from the Evoformer and transformed it into a molecule-level representation by averaging across atoms (Figure 4; pipeline schematic). For every resulting channel value, we computed Pearson’s R value for total hydrophobic surface area, molecular weight, number of hydrogen bond acceptors and donors, polar surface area, number of rotatable bonds, octanol-water partition coefficient, and number of aromatic rings; calculations were performed using the RDKit [16] and Mordred [26]. We carried out this computation for every molecule in the PDBBind test set (Figure 4; scatter plots). We found that some channels were significantly correlated with more than one property (Table SI.2, *p*-values were at least 10^−40^; we did not correct for multiple hypothesis testing as there were only 64 channels), indicating that QuickBind has in fact learned physicochemical characteristics of protein-ligand binding. Selecting the most strongly correlated property per channel, we found the number of H-bond acceptors and donors, the total hydrophobic surface area, and the number of rotatatable bonds to be the strongest features. We include additional results in section SI.9.

### 3.3 Case studies

To better understand QuickBind’s utility in real-world deployments, we predicted the bound poses of new ligands for five proteins in the PDBBind test set, which we selected based on their extensive experimental characterization and clinical significance (UniProt IDs B1MDI3, P56817, P17931, Q8ULI9, and P01116, Table SI.4). B1MDI3 is a tRNA guanine-methyltransferase, P56817 is BACE1, a beta-secretase relevant to the development of Alzheimer’s disease [27], P17931 is galectin-3, a galactose-specific lectin involved in cancer [28], Q8ULI9 is the human immunodeficiency virus 1 (HIV-1) protease, and P01116 is the GTPase K-Ras, a key cancer target. We used the protein crystal structures of the three lowest affinity binders from the PDBBind test set as input, and predicted the binding affinities and complex structures of all other compounds in the PDBBind test set. We treated all compounds not explicitly crystallized with the five target proteins as decoys (Figures SI.4-SI.6, Table SI.4). This likely resulted in some binders being mislabeled as non-binders.

We found QuickBind capable of discerning ligand binding characteristics across diverse ligand scaffolds and protein targets. Specifically, QuickBind distinguished binders from non-binders across various targets, including BACE1 (*p*-values of one-sided Wilcoxon rank sum tests of 0.0483, 0.0196, 0.0229), galectin-3 (0.0333, 0.0383, 0.0167), and K-Ras (< 0.00005, 0.0001, 0.0105), despite BACE1 and K-Ras binders having low average Tanimoto similarity. For BACE1 and galectin-3, QuickBind also accurately predicted poses with a ligand RMSD consistently below 2Å for the majority of ligands (success rates of 73% and 86%, respectively), based on our comparisons with PDBBind test set structures. For the HIV-1 protease, while the predicted binding affinities did not significantly differentiate binders from non-binders, QuickBind still excelled at predicting pose structures with high accuracy (all predictions below 2Å, Figure SI.7).

Our analyses also shed light on the limitations of QuickBind. It struggled when confronted with proteins whose conformation changes dramatically when binding target ligands versus low affinity ligands used as templates. For instance, KRAS structures had an average backbone RMSD of 7.7Å between input and true conformations, which resulted in a notable proportion of poses exhibiting high ligand RMSDs (only 17% of predictions fell below 5Å). For the wholly unseen tRNA guanine-methyltransferase, QuickBind struggled to predict significantly higher binding affinities for binders vs. non-binders and only predicted moderately accurate poses with a ligand RMSD below 5Å in 2% of cases.

## 4 Discussion

QuickBind is an ML model for blind molecular docking that optimizes runtime speed while retaining competitive pose prediction accuracy, providing a compelling option for high-throughput virtual screening. It furthermore captures physicochemical ligand properties known to influence molecular docking. Interpretability informs what aspects of the underlying physics a model has captured and may, if sufficiently well-developed, help guide the design of drug compounds. These considerations are important given the large costs involved in identifying and prioritizing compounds for experimental testing and optimization.

Our augmentation of QuickBind with affinity prediction capabilities make it suitable for identifying new ligands, which we showcase using multiple highly relevant drug targets. Except for TANKBind, existing blind docking and co-folding models do not predict binding affinities, and are therefore not applicable for screening applications. The combination of ligand pose and interaction strength can help in optimizing the potency and selectivity of drug candidates, although it remains to be seen how sensitive QuickBind is to minor structural changes, which can have profound effects on binding (so-called activity cliffs).

An added advantage of QuickBind’s formulation of binding affinity prediction is its lack of reliance on an experimental co-complex structure (unlike TANKBind), due to having been trained separately from the main docking model. Embeddings from any protein-ligand pair can be used, whether the structure is predicted or experimentally-derived. This means that QuickBind’s affinity module can be trained on BindingDB [29], a binding affinities database comprising millions of protein-ligand pairs, orders of magnitude more than structural complexes. Users can also rapidly finetune a binding affinity model for their own target proteins.

Evaluation using the PB Benchmark showed that QuickBind struggles to generate physically and chemically valid poses, but that many can be recovered by force field-based energy minimization. While energy minimization can increase model runtime, the binding affinity module can be used to first select a subset of promising compounds, whose poses can then be energy minimized.

## 5 Outlook

A major shortcoming of QuickBind and other ML-based molecular docking methods is their dependence on rigid re-docking, a task on which they are both trained and evaluated. In rigid re-docking, methods are provided with the *holo* protein structure that was originally co-crystallized with the query ligand, and the protein structure is not predicted. This does not reflect many real-world uses of molecular docking in which users only have access to the *apo* (unbound) structure or a *holo* structure co-crystallized with another ligand. Although QuickBind showed promise at cross-docking in the case studies we conducted, it did so only as long as the input protein did not deviate too much from the true structure. Ideally, models should be trained and evaluated in a flexible cross-docking setting, and in fact ML-based blind, flexible docking methods [30] have recently emerged. Future versions of QuickBind can be adapted into a flexible docking method capable of using *apo*, *holo*, or even predicted protein structures as input by updating protein residue frames as well as ligand frames. Alternatively, side chains can be made flexible by updating the rotational component of residue frames and side chain torsion angles.

Conceptually, one of our goals for QuickBind was to investigate how the AF2 architecture may be adapted to the task of docking and co-folding. While other docking [9, 10] and co-folding methods [4, 5, 6, 31] previously demonstrated that ideas and components from AF2 can be used in protein-ligand pose prediction, QuickBind uses essentially the entirety of AF2. This has provided multiple insights. First, it suggests that QuickBind could further benefit from AF2’s native confidence estimates and recycling, which were found to be important for AF2’s success. QuickBind could also benefit from more complicated, AF2-inspired loss functions, for example the structural violation loss which would likely lead to more PB-valid predictions. Second, QuickBind provides a rough estimate of how well an AF2-like model can perform molecular docking. The fact that it does not achieve state-of-the-art performance suggests that certain aspects of AF2 are not ideally suited for this task and anticipates some of the changes introduced in AF3, including a minimized MSA module and atomistic reasoning over ligands. Including further innovations from AF3 will likely result in an improved molecular docking tool (section SI.12). In turn, our findings also have implications for AF3. For instance, since QuickBind’s single representation captures physicochemical features of the ligand, it is likely that AF3’s single representation is highly information-dense as well, and may therefore be useful for tasks beyond pose prediction.

## Supplementary Material

Supplement 1

## References

[1] Kirkpatrick P. & Ellis C. Chemical space. Nature 432, 823 (2004).

[2] Ferreira L. G., Dos Santos R. N., Oliva G. & Andricopulo A. D. Molecular docking and structure-based drug design strategies. Molecules 20, 13384–13421 (2015). 26205061 10.3390/molecules200713384 PMC6332083

[3] Lyu J. Ultra-large library docking for discovering new chemotypes. Nature 566, 224–229 (2019). 30728502 10.1038/s41586-019-0917-9 PMC6383769

[4] KrishnaR. Generalized biomolecular modeling and design with rosettafold all-atom. Science 384, eadl2528 (2024).10.1126/science.adl252838452047

[5] Qiao Z., Nie W., Vahdat A., Miller T. F. & Anandkumar A. State-specific protein–ligand complex structure prediction with a multiscale deep generative model. Nature Machine Intelligence 6, 195–208 (2024).

[6] Bryant P., Kelkar A., Guljas A., Clementi C. & Noé F. Structure prediction of protein-ligand complexes from sequence information with umol. Nature Communications 15, 4536 (2024). 10.1038/s41467-024-48837-6 PMC11133481 38806453

[7] Abramson J. Accurate structure prediction of biomolecular interactions with alphafold 3. Nature 1–3 (2024). 10.1038/s41586-024-07487-w PMC11168924 38718835

[8] Stärk H., Ganea O., Pattanaik L., Barzilay R. & Jaakkola T. Equibind: Geometric deep learning for drug binding structure prediction. In International conference on machine learning, 20503–20521 (PMLR, 2022).

[9] Lu W. Tankbind: Trigonometry-aware neural networks for drug-protein binding structure prediction. Advances in neural information processing systems 35, 7236–7249 (2022).

[10] Zhang Y., Cai H., Shi C., Zhong B. & Tang J. E3bind: An end-to-end equivariant network for protein-ligand docking. arXiv preprint arXiv:2210.06069 (2022).

[11] Krivák R. & Hoksza D. P2rank: machine learning based tool for rapid and accurate prediction of ligand binding sites from protein structure. Journal of Cheminformatics 10, 39 (2018). 30109435 10.1186/s13321-018-0285-8 PMC6091426

[12] Jumper J. Highly accurate protein structure prediction with alphafold. Nature 596, 583–589 (2021). 34265844 10.1038/s41586-021-03819-2 PMC8371605

[13] Pei Q. Fabind: Fast and accurate protein-ligand binding. Advances in Neural Information Processing Systems 36 (2024).

[14] Corso G., Stärk H., Jing B., Barzilay R. & Jaakkola T. Diffdock: Diffusion steps, twists, and turns for molecular docking. arXiv preprint arXiv:2210.01776 (2022).

[15] Lin Z. Language models of protein sequences at the scale of evolution enable accurate structure prediction. BioRxiv 2022, 500902 (2022).

[16] Landrum G. Rdkit: Open-source cheminformatics. URL https://www.rdkit.org.

[17] Liu Z. Forging the basis for developing protein–ligand interaction scoring functions. Accounts of Chemical Research 50, 302–309 (2017). 28182403 10.1021/acs.accounts.6b00491

[18] Buttenschoen M., Morris G. M. & Deane C. M. Posebusters: Ai-based docking methods fail to generate physically valid poses or generalise to novel sequences. Chemical Science 15, 3130–3139 (2024). 38425520 10.1039/d3sc04185a PMC10901501

[19] Alhossary A., Handoko S. D., Mu Y. & Kwoh C.-K. Fast, accurate, and reliable molecular docking with quickvina 2. Bioinformatics 31, 2214–2216 (2015). 25717194 10.1093/bioinformatics/btv082

[20] Hassan N. M., Alhossary A. A., Mu Y. & Kwoh C.-K. Protein-ligand blind docking using quickvina-w with inter-process spatio-temporal integration. Scientific Reports 7, 15451 (2017). 29133831 10.1038/s41598-017-15571-7 PMC5684369

[21] McNutt A. T. Gnina 1.0: molecular docking with deep learning. Journal of Cheminformatics 13, 43 (2021). 34108002 10.1186/s13321-021-00522-2 PMC8191141

[22] Meli R. & Biggin P. C. spyrmsd: symmetry-corrected rmsd calculations in python. Journal of Cheminformatics 12, 49 (2020). 33431033 10.1186/s13321-020-00455-2 PMC7457508

[23] Floristean C., Bouatta N. & AlQuraishi M. Unpublished Work.

[24] Kabsch W. A solution for the best rotation to relate two sets of vectors. Acta Crystallographica Section A: Crystal Physics, Diffraction, Theoretical and General Crystallography 32, 922–923 (1976).

[25] Lyu J. Ultra-large library docking for discovering new chemotypes. Nature 566, 224–229 (2019). 30728502 10.1038/s41586-019-0917-9 PMC6383769

[26] Moriwaki H., Tian Y.-S., Kawashita N. & Takagi T. Mordred: a molecular descriptor calculator. Journal of Cheminformatics 10, 4 (2018). 29411163 10.1186/s13321-018-0258-y PMC5801138

[27] Kitazume S. Alzheimer’s *β*-secretase, *β*-site amyloid precursor protein-cleaving enzyme, is responsible for cleavage secretion of a golgi-resident sialyltransferase. Proceedings of the National Academy of Sciences 98, 13554–13559 (2001). 10.1073/pnas.241509198 PMC61079 11698669

[28] Li S., Pritchard D. M. & Yu L.-G. Galectin-3 promotes secretion of proteases that decrease epithelium integrity in human colon cancer cells. Cell Death & Disease 14, 268 (2023). 37055381 10.1038/s41419-023-05789-x PMC10102123

[29] Gilson M. K. Bindingdb in 2015: a public database for medicinal chemistry, computational chemistry and systems pharmacology. Nucleic acids research 44, D1045–D1053 (2016). 26481362 10.1093/nar/gkv1072 PMC4702793

[30] Lu W. Dynamicbind: predicting ligand-specific protein-ligand complex structure with a deep equivariant generative model. Nature Communications 15, 1071 (2024). 10.1038/s41467-024-45461-2 PMC10844226 38316797

[31] Nakata S., Mori Y. & Tanaka S. End-to-end protein–ligand complex structure generation with diffusion-based generative models. BMC Bioinformatics 24, 233 (2023). 37277701 10.1186/s12859-023-05354-5 PMC10240776

[32] Evans R. Protein complex prediction with alphafold-multimer. biorxiv 2021–10 (2021).

[33] Paszke A. Pytorch: An imperative style, high-performance deep learning library. Advances in neural information processing systems 32 (2019).

[34] Falcon W. & The PyTorch Lightning team. PyTorch Lightning (2019). URL https://github.com/Lightning-AI/lightning.

[35] Ahdritz G. Openfold: Retraining alphafold2 yields new insights into its learning mechanisms and capacity for generalization. Nature Methods 1–11 (2024). 38744917 10.1038/s41592-024-02272-z PMC11645889

[36] Loshchilov I. & Hutter F. Decoupled weight decay regularization. arXiv preprint arXiv:1711.05101 (2017).

[37] Kingma D. P. & Ba J. Adam: A method for stochastic optimization. arXiv preprint arXiv:1412.6980 (2014).

[38] Nguyen H., Case D. A. & Rose A. S. Nglview–interactive molecular graphics for jupyter notebooks. Bioinformatics 34, 1241–1242 (2018). 29236954 10.1093/bioinformatics/btx789 PMC6031024

[39] Chen L. Transformercpi: improving compound–protein interaction prediction by sequence-based deep learning with self-attention mechanism and label reversal experiments. Bioinformatics 36, 4406–4414 (2020). 32428219 10.1093/bioinformatics/btaa524

[40] Li S. Monn: a multi-objective neural network for predicting compound-protein interactions and affinities. Cell Systems 10, 308–322 (2020).

[41] Moon S., Zhung W., Yang S., Lim J. & Kim W. Y. Pignet: a physics-informed deep learning model toward generalized drug–target interaction predictions. Chemical Science 13, 3661–3673 (2022). 35432900 10.1039/d1sc06946b PMC8966633

[42] Jiang D. Interactiongraphnet: A novel and efficient deep graph representation learning framework for accurate protein–ligand interaction predictions. Journal of Medicinal Chemistry 64, 18209–18232 (2021). 34878785 10.1021/acs.jmedchem.1c01830

[43] Somnath V. R., Bunne C. & Krause A. Multi-scale representation learning on proteins. Advances in Neural Information Processing Systems 34, 25244–25255 (2021).

[44] Wang P. Structure-aware multimodal deep learning for drug–protein interaction prediction. Journal of Chemical Information and Modeling 62, 1308–1317 (2022). 35200015 10.1021/acs.jcim.2c00060

[45] Rogers D. & Hahn M. Extended-connectivity fingerprints. Journal of Chemical Information and Modeling 50, 742–754 (2010). 20426451 10.1021/ci100050t

