# Supplementary material for: QuickBind: A Light-Weight And Interpretable Molecular Docking Model
Source: ArXiv. 2024 Oct 21:arXiv:2410.16474v1. Preprint. [Version 1] (PMC11537332)
Supplement: Supplement 1 [file NIHPP2410.16474v1-supplement-1.pdf]

## Supplementary Information

## SI.1 Algorithms

**Algorithm 1** QUICKBIND architecture. Module names correspond to the names of the algorithms in the supplementary information of [12] and the same notation is used. The Evoformer Stack does not contain the MSAColumnAttention module. New modules are highlighted in blue. Concatenation (concat) and deconcatenation (deconcat) happens along the sequence and atom dimension, unless otherwise stated. $\{r_i\}$, protein features. $\{l_i\}$, ligand features. $\{\vec{x}_{C_\alpha}\}$, $\{\vec{x}_{C}\}$, $\{\vec{x}_{N}\}$, coordinates of $C_\alpha$, C, and N atoms. $\left\{\vec{t}_i^{\text{lig}}\right\}$, initial ligand coordinates. $\{f_i^{\text{res\_index}}\}$, indices of amino acid residues. $\left\{f_{ij}^{\text{adj}}\right\}$, ligand adjacency matrix.

**function** QUICKBIND($\{r_i\}, \{l_i\}, \{\vec{x}_{C_\alpha}\}, \{\vec{x}_{C}\}, \{\vec{x}_{N}\}, \left\{\vec{t}_i^{\text{lig}}\right\}, \{f_i^{\text{res\_index}}\}, \left\{f_{ij}^{\text{adj}}\right\}$)

  $\left\{\vec{X}_{C}^{\text{pseudo}}\right\}, \left\{\vec{X}_{N}^{\text{pseudo}}\right\} \leftarrow \text{getadjacentatoms}\left(\left\{\vec{t}_i^{\text{lig}}\right\}\right)$  # as described in Methods

  $T_i^{\text{lig}} \leftarrow \text{rigidFrom3Points}\left(\vec{t}_i^{\text{lig}}, \vec{x}_{C}^{\text{pseudo}}, \vec{x}_{N}^{\text{pseudo}}\right)$

  $T_i^{\text{prot}} \leftarrow \text{rigidFrom3Points}\left(\vec{x}_{C_\alpha}, \vec{x}_{C}, \vec{x}_{N}\right)$

  $\{T_i\} \leftarrow \text{concat}\left(\{T_i^{\text{prot}}\}, \left\{T_i^{\text{lig}}\right\}\right)$

  $\{s_i\}, \{z_{ij}\} \leftarrow \text{InputEmbedder}\left(\{r_i\}, \{l_i\}, \{\vec{x}_{C_\alpha}\}, \left\{\vec{t}_i^{\text{lig}}\right\}, \{f_i^{\text{res\_index}}\}, \left\{f_{ij}^{\text{adj}}\right\}\right)$

  # Evoformer

  $\{s_i\}, \{z_{ij}\} \leftarrow \text{EvoformerStack}\left(\{s_i\}, \{z_{ij}\}\right)$

  # Structure Module

  $s_i \leftarrow \text{LayerNorm}(s_i)$

  $z_{ij} \leftarrow \text{LayerNorm}(z_{ij})$

  $s_i \leftarrow \text{Linear}(s_i)$

  **for all** $l \in [1, \dots, N_{\text{Struct}}]$ **do**

    $\{s_i\} \mathrel{+}= \text{InvariantPointAttention}\left(\{s_i\}, \{z_{ij}\}, \{T_i\}\right)$

    $s_i \leftarrow \text{LayerNorm}\left(\text{Dropout}_{0.1}(s_i)\right)$

    $s_i \leftarrow s_i + \text{Linear}(\text{relu}(\text{Linear}(\text{relu}(\text{Linear}(s_i)))))$

    $s_i \leftarrow \text{LayerNorm}\left(\text{Dropout}_{0.1}(s_i)\right)$

    $\{T_i^{\text{prot}}\}, \left\{T_i^{\text{lig}}\right\} \leftarrow \text{deconcat}\left(\{T_i\}\right)$

    $T_i^{\text{lig}} \leftarrow T_i^{\text{lig}} \circ \text{BackboneUpdate}(s_i)$  # Use predicted quaternion and

    $\{T_i\} \leftarrow \text{concat}\left(\{T_i^{\text{prot}}\}, \left\{T_i^{\text{lig}}\right\}\right)$  # translation to update ligand frames

  **end for**

  $\left\{\mathbf{R}_i^{\text{lig}}\right\}, \left\{\vec{t}_i^{\text{lig}}\right\} = \left\{T_i^{\text{lig}}\right\}$

  **return** $\left\{\vec{t}_i^{\text{lig}}\right\}$

**end function**

**Algorithm 2** Algorithm for the generation of input embeddings.

**function** INPUTEMBEDDER($\{r_i\}, \{l_i\}, \{\vec{x}_{C_\alpha}\}, \left\{\vec{t}_i^{\text{lig}}\right\}, \{f_i^{\text{res\_index}}\}, \left\{f_{ij}^{\text{adj}}\right\}$)

$r_i \leftarrow \text{Linear}(r_i)$

$l_i \leftarrow \text{Linear}(l_i)$

$\{s_i\} \leftarrow \text{concat}(\{r_i\}, \{l_i\})$

$a_i \leftarrow \text{Linear}(s_i)$

$b_i \leftarrow \text{Linear}(s_i)$

$z_{ij} \leftarrow a_i + b_j$

$\{p_{ij}\} \leftarrow \text{relpos}\left(\{f_i^{\text{res\_index}}\}\right)$

$q_{ij} \leftarrow \text{Linear}\left(f_{ij}^{\text{adj}}\right)$

$\{\omega_{ij}\} \leftarrow \text{blockdiag}(\{p_{ij}\}, \{q_{ij}\})$ # combine into a blockdiagonal tensor

$d_{ij}^{\text{prot}} \leftarrow \text{Linear}\left(\left\|\vec{x}_{C_\alpha}^{i} - \vec{x}_{C_\alpha}^{j}\right\|_2\right)$

$d_{ij}^{\text{lig}} \leftarrow \text{Linear}\left(\left\|\vec{t}_i^{\text{lig}} - \vec{t}_j^{\text{lig}}\right\|_2\right)$

$\{d_{ij}\} \leftarrow \text{blockdiag}\left(\{d_{ij}^{\text{prot}}\}, \left\{d_{ij}^{\text{lig}}\right\}\right)$ # combine into a blockdiagonal tensor

$z_{ij} = z_{ij} + \omega_{ij} + d_{ij}$

$s_i \leftarrow \text{Linear}(s_i)$

**return** $\{s_i\}, \{z_{ij}\}$

**end function**

**Algorithm 3** Algorithm for updating the ligand frames.

**function** BACKBONEUPDATE($\{s_i\}$)

$\{s_i^{\text{prot}}\}, \left\{s_i^{\text{lig}}\right\} \leftarrow \text{deconcat}(\{s_i\})$

$m_i \leftarrow \text{MultiHeadAttention}\left(q = \left\{s_i^{\text{lig}}\right\}, k = \{s_i^{\text{prot}}\}, v = \{s_i^{\text{prot}}\}\right)$

$s_i^{\text{lig}} \mathrel{+}= m_i$

$b_i, c_i, d_i, \vec{t}_i \leftarrow \text{Linear}\left(s_i^{\text{lig}}\right)$ # quaternion and translation

**return** $b_i, c_i, d_i, \vec{t}_i$

**end function**

**Algorithm 4** Algorithm for getting the coordinates of a dummy atom when an atom has only one neighbor. Find the vector with the same $x$ and $y$ coordinates as the bond vector between the atom under question and its one neighbor, such that the dot product of the two vanishes, and subtract that vector from the coordinates of the given atom. $\vec{x}_{\text{C}}$, coordinates of atom under consideration, $\vec{x}_{\text{N}}$, coordinates of its neighbor.

**function** GETDUMMYATOMCOORDS($\vec{x}_{\text{C}}, \vec{x}_{\text{N}}$)

$\vec{b} \leftarrow \vec{x}_{\text{C}} - \vec{x}_{\text{N}}$

$x_b, y_b, z_b \leftarrow \vec{b}$ # get $x$, $y$, $z$ coordinates

$z_b' \leftarrow -\dfrac{x_b^2 + y_b^2}{z_b}$

$\vec{b}' \leftarrow \begin{pmatrix} x_b \\ y_b \\ z_b' \end{pmatrix}$ # $\vec{b} \cdot \vec{b}' = 0$

$\vec{x}_{\text{N}'} \leftarrow \vec{x}_{\text{C}} - \vec{b}'$

**return** $\vec{x}_{\text{N}'}$

**end function**

**Algorithm 5** Binding affinity prediction model.

**function** BINDINGAFFINITYPREDICTOR($\{s_i\}$)
  $s_i \leftarrow \mathrm{LayerNorm}\left(s_i\right)$
  $s_i \leftarrow \mathrm{Linear}\left(\mathrm{silu}\left(\mathrm{Linear}\left(s_i\right)\right)\right)$
  $a \leftarrow \mathrm{mean}\left(\{s_i\}\right)$ # along sequence dimension
  $a \leftarrow \mathrm{Linear}\left(\mathrm{relu}\left(\mathrm{Linear}\left(\mathrm{relu}\left(\mathrm{Linear}\left(a\right)\right)\right)\right)\right)$ # $\mathbb{R}^{64} \rightarrow \mathbb{R}^{32} \rightarrow \mathbb{R}$
  **return** $a$
**end function**

## SI.2 Protein and ligand features

Residue types are one-hot encoded. Ligand atoms have the following features: atomic number (H, C, N, O, F, P, S, Cl, Br, I, other), chirality, degree (1 through 4, or other), formal charge (-1, 0, 1, or other), number of connected H atoms (0 through 3, or other), hybridization, presence in a ring, and presence in an aromatic ring. Early versions included atomic numbers 1 through 119, degrees up to 10, formal charges of -5 through 5, implicit valence, number of connected hydrogens of up to 8, number of radical electrons, hybridization, presence in an aromatic ring, the number of rings it is in, presence in a ring of size 3, 4, 5, 6, 7, or 8, similar to ref. [8]. These additional features did not improve performance and so were omitted. Ligand coordinates are initialized using a random RDKit conformer [16].

## SI.3 Cropping

To reduce QUICKBIND's memory footprint during training, input protein sequences were cropped to 512 or 256 residues for models without and with the Evoformer module, respectively. Since model performance drops noticeably for shorter crop sizes, the final model was finetuned with a crop size of 512 residues. At inference time, the full protein sequence is used. Depending on available GPU memory, inference might therefore have to be run on CPUs. Using our resources, we had to restrict inference on GPUs to proteins shorter than 2,000 residues. For multi-chain proteins, sequences were concatenated in the order in which they appear in the PDB file.

We tested different cropping strategies: random contiguous cropping; setting the residue whose $C_\alpha$ atom is closest to any ligand atom as the midpoint of the contiguous cropped fragment (binding site cropping, BSC); selecting the $x$ residues closest to any ligand atom (spatial cropping), and a setting in which the protein was cropped randomly or spatially with a probability of 0.5.

## SI.4 Hyperparameter screening

Because extensive hyperparameter screening of the full QUICKBIND model would have been too computationally expensive, we optimized many hyperparameters and architectural choices using two smaller variants, both trained without ligand frames and still updating H atom positions:

- QUICKBIND-S, which lacks the Evoformer module and contains just four or eight Structure module blocks with unshared weights, trained without batching.
- QUICKBIND-M, which lacks Triangle Attention in the Evoformer stack (its most expensive module), trained with a batch size of 12 or 16.

The main results of the hyperparameter screening are summarized in Table SI.1. Furthermore, several ways to generate the ligand coordinate updates from the final single representation were tested. In general, the final single representation is separated into a protein and a ligand single representation. Then, either:

- only the ligand single representation is passed through a linear layer to produce the coordinate updates, or
- the protein single representation is summed along the sequence dimension or the corresponding mean is taken, and this pooled protein representation is concatenated with the ligand single representation before passing it through the linear layer, or

- the outer product of the protein and ligand single representations is summed along the sequence dimension or the corresponding mean is taken, and then passed through the linear layer, or
- the output of an attention layer with the query vectors coming from the ligand and key and value vectors coming from the protein are concatenated with the ligand single representation and passed through the final linear layer.

The last approach led to the best results.

We also tested scaling the coordinate update by a factor of 10, as is done in AF2, but did not find this to improve model performance. Furthermore, we briefly experimented with first applying a global rototranslation of the ligand coordinates, and then either finetuning all ligand coordinates or just changing the torsion angles of rotatable bonds, but did not find this to lead to better results. Using a gated variant of the IPA module [23] improved model performance compared to the standard IPA module. In addition, we also tested two ideas from ALPHAFOLD-MULTIMER (AF-MULTIMER) [32], moving the outer product mean to the beginning of the Evoformer block and the multimer version of the relative positional encoding, but neither improved model performance.

## SI.5 Training details

QUICKBIND was implemented using PyTorch [33], PyTorch Lightning [34], OpenFold [35], and the RDKit [16]. Models were trained using the AdamW optimizer [36] with a learning rate and a weight decay coefficient of $10^{-4}$, early stopping with a patience of 50 epochs, and a batch size of 16. The binding affinity prediction model was trained using the Adam optimizer [37] with a learning rate of 0.01, early stopping with a patience of 50 epochs, and a batch size of 64 using a mean squared error (MSE) loss. The model weights with the best performance on the validation set were chosen for evaluation on the test set. Training the final QUICKBIND model took several weeks on eight NVIDIA A40 GPUs, but QUICKBIND-S and QUICKBIND-M variants were trained in two weeks or less. The final model was trained with 5 different seeds. Some replicas got stuck in local minima (success rates of 0.0%, 2.5%, 12.1%, 15.4%, 23.4%), and only the best-performing model was finetuned with a crop size of 512. It is plausible that models that got stuck in local minima would have reached a similar performance to the final model after the finetuning stage, but we did not test this because the training time when including triangle attention scales very unfavorably with sequence length. Force-field minimization was performed as described in ref. [18] using a script kindly provided by one of the authors. Visualizations were generated using NGLview [38].

Table SI.1: Results of hyperparameter search. Accepted configurations are indicated by ✓or underlined, rejected configurations are indicated by ✗. $\mathcal{L}_{\mathrm{MSE}}$ - Mean squared error loss. $\mathcal{L}_{\mathrm{centroid}}$ - Centroid loss. $\mathcal{L}_{\mathrm{Kabsch}}$ - Kabsch loss. $\mathcal{L}_{\mathrm{FAPE}}$ - FAPE loss. $\mathcal{L}_{\mathrm{FAPE}}$ was tested with a $\mathcal{L}_{\mathrm{FAPE}}^{\mathrm{prot-lig}}$ clamped at 10 Å and without clamping. $\mathcal{L}_{\mathrm{FAPE}}^{\mathrm{aux}}$ - intermediate FAPE losses acting on the outputs of every Structure module block. $\mathcal{L}_{\mathrm{dist}}^{\mathrm{lig-lig}}$ - ligand distogram loss head, a cross-entropy loss that acts on a symmetrized version of the ligand pair representation, similar to AF2, with 42 distance bins between 1 and 5 Å. $\mathcal{L}_{\mathrm{dist}}^{\mathrm{prot-lig}}$ - protein-ligand distogram loss head using the symmetrized off-diagonal parts of the pair representation, using the same bins as in AF2. $\mathcal{L}_{\mathrm{torsion}}$ - torsion angle loss. Black hole initialization refers to collapsing all ligand atoms at the origin, as is done in AF2.

| | |
|---|---|
| **Input embeddings** | |
| Pairwise distances | ✓ |
| Radial basis projection of pairwise distances | ✗ |
| AF2 relative positional encoding | ✓ |
| AF2-MULTIMER relative positional encoding | ✗ |
| Adjacency matrix | ✓ |
| ... w/ one-hot encoded bond types | ✗ |
| ... w/ topological distance | ✗ |
| **Loss function** | |
| $\mathcal{L}_{\mathrm{MSE}}$, $\mathcal{L}_{\mathrm{centroid}}$, $\underline{\mathcal{L}_{\mathrm{Kabsch}}}$, $\underline{\mathcal{L}_{\mathrm{FAPE}}}$ (clamped and $\underline{\text{unclamped}}$) , $\underline{\mathcal{L}_{\mathrm{FAPE}}^{\mathrm{aux}}}$, $\mathcal{L}_{\mathrm{dist}}^{\mathrm{lig-lig}}$, $\mathcal{L}_{\mathrm{dist}}^{\mathrm{prot-lig}}$, $\mathcal{L}_{\mathrm{torsion}}$ | |
| **Cropping** | |
| Random | ✗ |
| Binding site cropping | ✓ |
| Spatial cropping | ✗ |
| Random and spatial cropping | ✗ |
| **Ligand frames** | |
| Keeping the rotation matrix fixed | ✗ |
| Updating the rotation matrix | ✓ |
| **Ligand initialisation** | |
| At origin | ✓ |
| Black hole initialisation | ✗ |
| Randomly translated and rotated | ✗ |
| **Structure module** | |
| Number of blocks | 4, $\underline{8}$ |
| **Evoformer** | |
| Number of blocks | 8, $\underline{12}$ |
| Number of MSA attention heads | $\underline{8}$, 12 |

## SI.6 Exemplary predictions

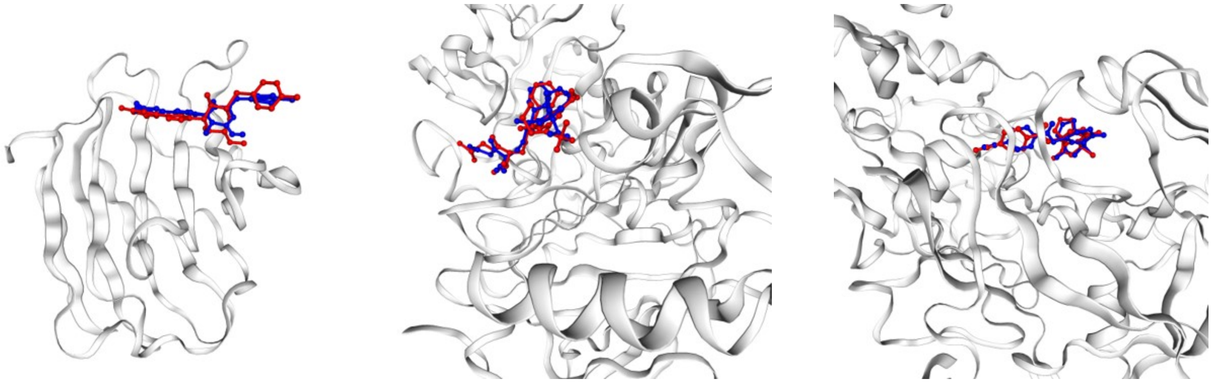

(a) 1.8Å. (b) 1.1Å. (c) 0.7Å.

Figure SI.1: Three examples of QUICKBIND predictions and their RMSDs, randomly chosen from 100 lowest-RMSD predictions on the PDBBind test set. The ground-truth ligand is shown in red, the QUICKBIND prediction is shown in blue.

## SI.7 PB failure modes

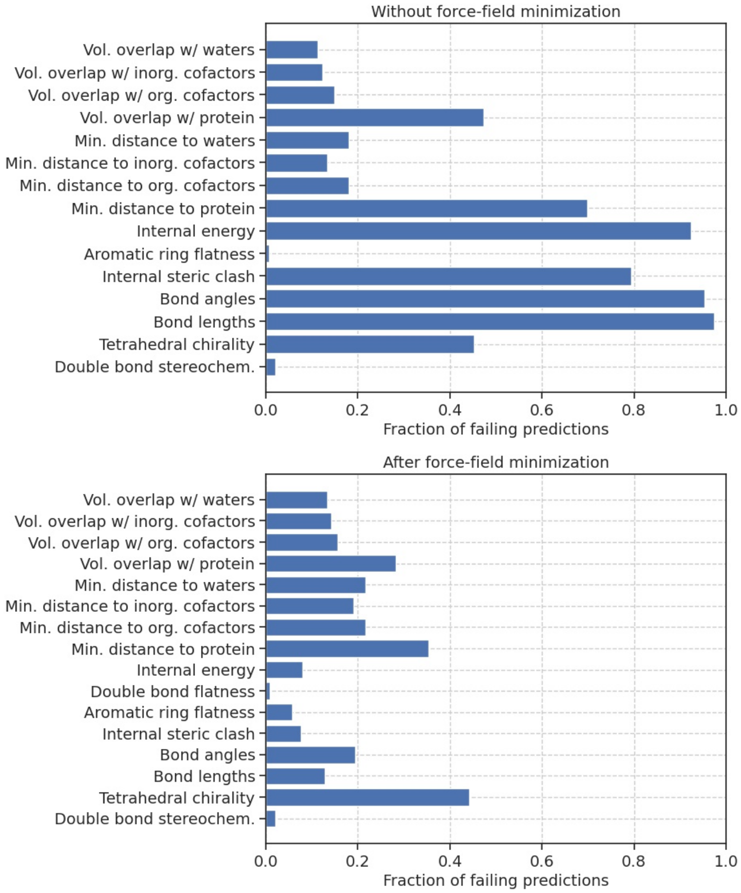


Figure SI.2: Fraction of all QUICKBIND predictions on the PB test set that fail PB tests, before and after force field minimization.

## SI.8 Correlation with physicochemical features

Table SI.2: Correlation between physicochemical features of the ligand and mean channel values of the molecule representation. The magnitude of the Pearson's $R$ values for the octanol-water partition coefficient and number of aromatic rings was less than 0.6.

| Feature | Pearson's $R$ | $p$-value | Channel |
|---|---|---|---|
| Total hydrophobic surface area | -0.72 | $10^{-59}$ | 57 |
| Molecular weight | 0.73 | $10^{-62}$ | 27 |
| Number of hydrogen bond acceptors | 0.63 | $10^{-42}$ | 11 |
| Number of hydrogen bond donors | 0.70 | $10^{-54}$ | 31 |
| Polar surface area | 0.71 | $10^{-56}$ | 27 |
| Number of rotatable bonds | 0.76 | $10^{-70}$ | 27 |

## SI.9 Additional interpretability studies

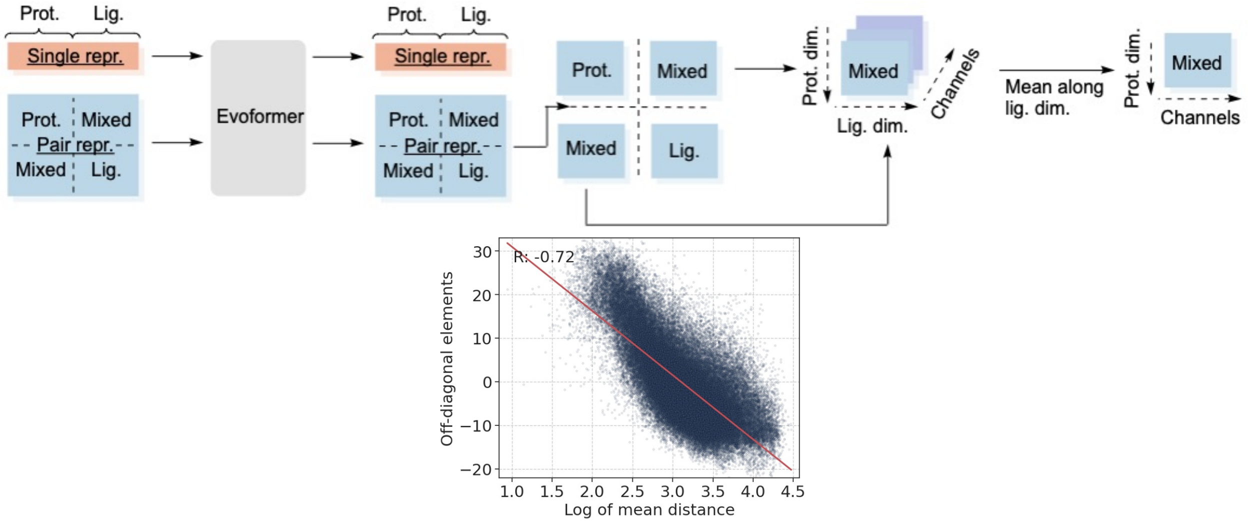


Figure SI.3: The off-diagonal elements of the pair representation contain information about the final ligand atom positions relative to the protein. The off-diagonal elements of the pair representation after the Evoformer block were symmetrized and the mean along the ligand dimension was taken. Some channel values correlate with the logarithm of the mean $C_\alpha$-ligand atom distance; for one particular channel the Pearson's R value is -0.72 at a $p$-value smaller than machine precision.

We wanted to understand how QUICKBIND obtains its initial guess of the docked ligand pose. The pair representation contains a protein and a ligand block, as well as mixed off-diagonal elements. Among other features, the protein and ligand blocks are constructed from the pairwise distances of the $C_\alpha$ and ligand atoms, respectively, but the off-diagonal elements do not contain any spatial information. We hypothesized that the model would use these off-diagonal elements for information about the interaction of the protein and the ligand, including an initial guess about their pairwise distances. We therefore took the off-diagonal elements of the pair representation after the Evoformer block, symmetrised them by transposing the lower off-diagonal block and computing the element-wise mean, and finally took the mean along the ligand dimension to obtain an $N_{C_\alpha} \times c$ dimensional matrix, where $N_{C_\alpha}$ is the number of $C_\alpha$ atoms and $c$ is the hidden channel dimension (Figure SI.3). Indeed, we found that already the mean along the channel dimension is weakly correlated with the logarithm of the mean $C_\alpha$-ligand atom distance with a Pearson's R value of -0.53. This correlation is much stronger for some channels. In particular, there is a channel that correlates with the logarithm of the mean $C_\alpha$-ligand atom distance with a Pearson's R value of -0.72.

## SI.10 Binding affinity prediction

Table SI.3: Root-mean-square error (RMSE), Pearson correlation coefficient (PCC), Spearman's rank correlation coefficient (SRCC), and mean absolute error (MAE) of QuickBind and other methods for binding affinity prediction on the PDBBind test set, computed using the mean and standard deviation across three runs. All methods predict negative log-transformed binding affinities. Values for other methods are taken from ref. [9].

| Method | RMSE ↓ | PCC ↑ | SRCC ↑ | MAE ↓ |
|---|---|---|---|---|
| TransformerCPI [39] | $1.741 \pm 0.058$ | $0.576 \pm 0.022$ | $0.540 \pm 0.016$ | $1.404 \pm 0.040$ |
| MONN [40] | $1.438 \pm 0.027$ | $0.624 \pm 0.037$ | $0.589 \pm 0.011$ | $1.143 \pm 0.052$ |
| PIGNet [41] | 2.64 | 0.51 | 0.49 | 2.1 |
| IGN [42] | $1.433 \pm 0.028$ | $0.698 \pm 0.007$ | $0.641 \pm 0.014$ | $1.169 \pm 0.036$ |
| HOLOPROT [43] | $1.546 \pm 0.065$ | $0.602 \pm 0.006$ | $0.571 \pm 0.018$ | $1.208 \pm 0.038$ |
| STAMPDPI [44] | 1.658 | 0.545 | 0.411 | 1.325 |
| TANKBind [9] | $1.346 \pm 0.007$ | $0.726 \pm 0.007$ | $0.703 \pm 0.017$ | $1.070 \pm 0.019$ |
| QuickBind | $1.577 \pm 0.011$ | $0.548 \pm 0.025$ | $0.482 \pm 0.024$ | $1.292 \pm 0.008$ |

## SI.11 Virtual screening results

Table SI.4 summarizes important characteristics of the five proteins in the PDBBind test set with the highest number of complex structures in the PDBBind test set, as well as QUICKBIND's cross-docking performance. In particular, it contains:

- the number of complex structures in the PDBBind test set (# Binders),
- the number of complex structures in the PDBBind train set (# Train ex.),
- the average $C_\alpha$ RMSD between input and true protein structures (BB RMSD),
- and the average Tanimoto similarity between the lowest, second-lowest, or third-lowest affinity binder and the remaining binders, calculated using extended-connectivity fingerprints [45] with a radius of 3 (TS).

We evaluate QUICKBIND's cross-docking performance using the fraction of predictions with a ligand RMSD below 2Å (% < 2Å) and the fraction of predictions with a ligand RMSD below 5Å (% < 5Å), after aligning the $C_\alpha$ atoms of the input and the true protein structure using the Kabsch algorithm. For this alignment and for calculating BB RMSD we only consider $C_\alpha$ atoms that were successfully extracted for both complexes. Where applicable, we provide the mean and standard deviation across the three runs with the crystal structures of the lowest, second-lowest, and third-lowest affinity binder. For all proteins, we tested if the predicted binding affinities of binders were higher than those of non-binders using one-sided Wilcoxon rank-sum tests.

Table SI.4: Characteristics of the five proteins in the PDBBind test set with the highest number of complex structures and QUICKBIND's cross-docking performance.

| UniProt ID | # Binders | # Train ex. | % < 2 Å | % < 5 Å | BB RMSD [Å] | TS |
|---|---|---|---|---|---|---|
| B1MDI3 | 19 | 0 | $0.0 \pm 0.0$ | $2 \pm 3$ | $0.36 \pm 0.09$ | $0.26 \pm 0.08$ |
| P56817 | 16 | 308 | $73 \pm 0$ | $93 \pm 0$ | $0.96 \pm 0.04$ | $0.209 \pm 0.005$ |
| P17931 | 15 | 22 | $86 \pm 0$ | $93 \pm 0$ | $0.21 \pm 0.07$ | $0.620 \pm 0.029$ |
| Q8ULI9 | 14 | 3 | $100 \pm 0$ | $100 \pm 0$ | $0.15 \pm 0.01$ | $0.64 \pm 0.10$ |
| P01116 | 13 | 8 | $0.0 \pm 0.0$ | $17 \pm 12$ | $7.7 \pm 2.7$ | $0.101 \pm 0.026$ |

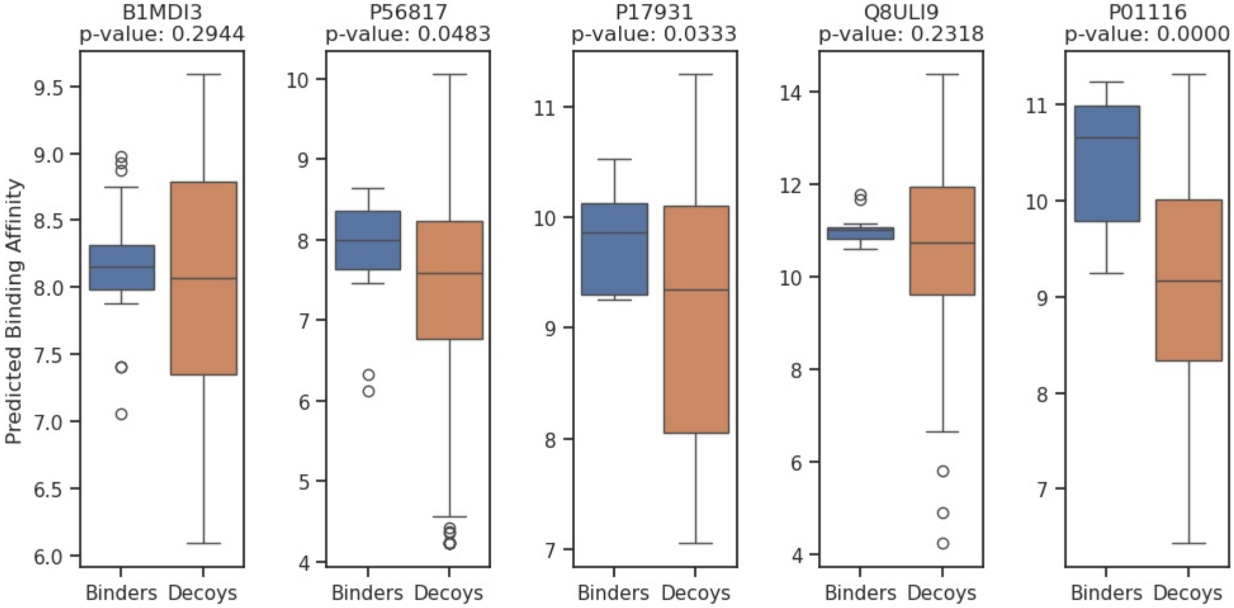


Figure SI.4: Predicted binding affinities of true binders and decoys for the five proteins in the PDBBind test set with the highest number of binders, using the protein structure of the lowest affinity binder.

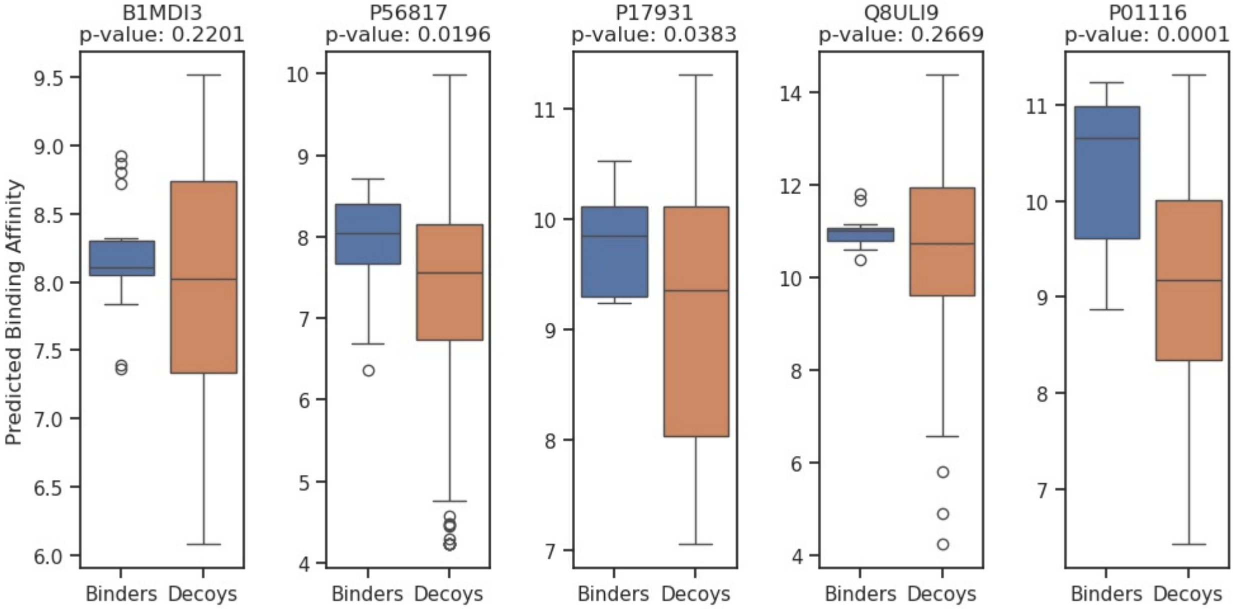


Figure SI.5: Predicted binding affinities of true binders and decoys for the five proteins in the PDBBind test set with the highest number of binders, using the protein structure of the second-lowest affinity binder.

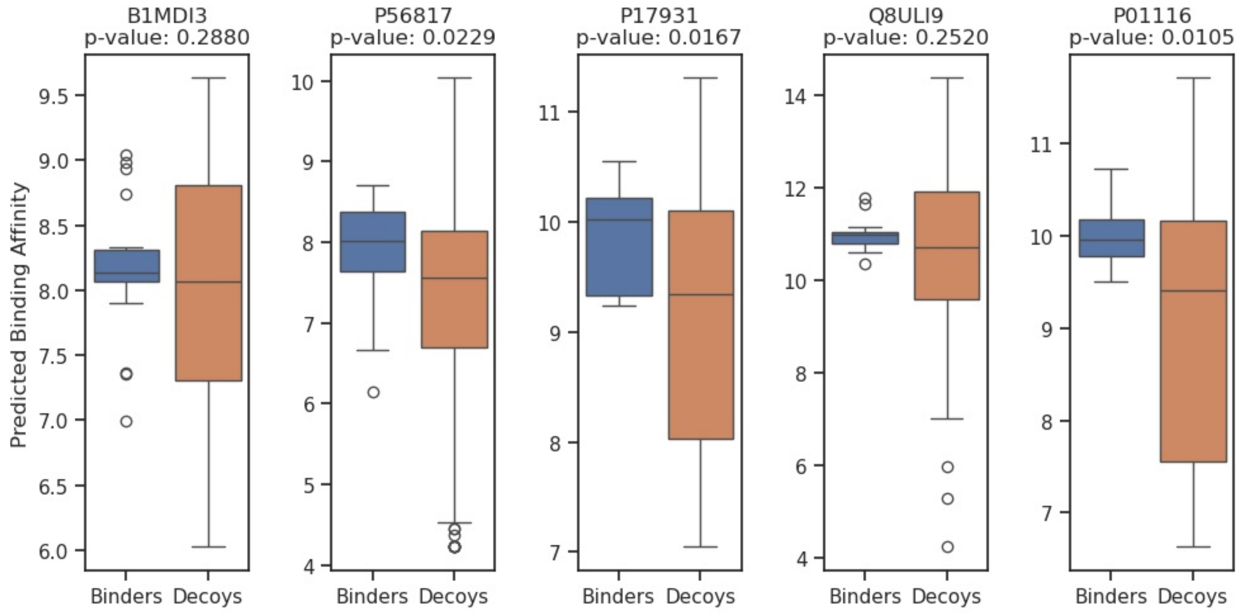


Figure SI.6: Predicted binding affinities of true binders and decoys for the five proteins in the PDBBind test set with the highest number of binders, using the protein structure of the third-lowest affinity binder.

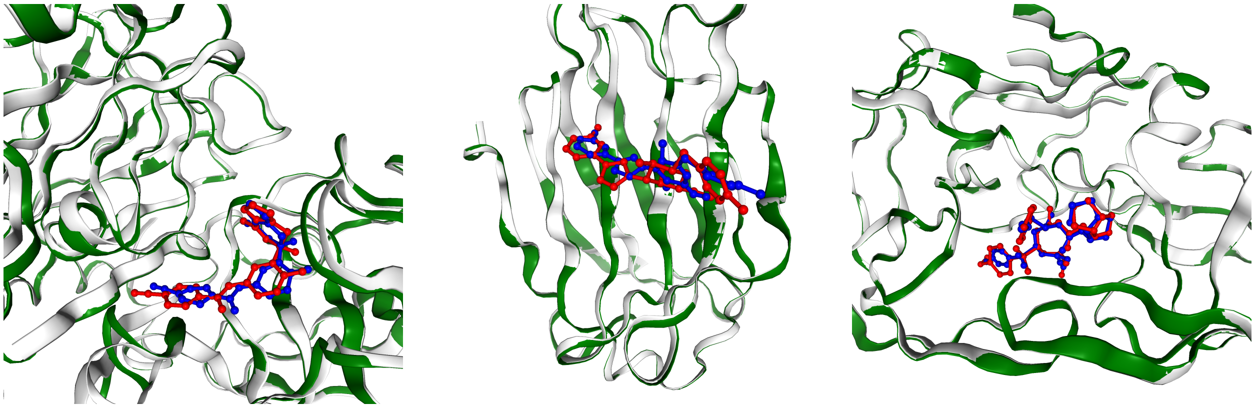

(a) P56817, 0.8Å. (b) P17931, 1.1Å. (c) Q8ULI9, 0.7Å.

Figure SI.7: Lowest RMSD QuickBind cross-docking predictions for P56817, P17931, and Q8ULI9. The ground-truth ligand and protein are shown in red and white, respectively. The input protein structure is shown in green and the QuickBind prediction is shown in blue.

## SI.12 Retrospective comparison with AF3

In this section we discuss our observations on the differences introduced to AF2 by QuickBind versus those made by AF3.

First, AF3 no longer uses residue reference frames and eschews SE(3)-equivariance entirely. In early internal experiments on QuickBind, we similarly observed higher success rates when ligand reference frames were omitted. We opted to include them as the model would otherwise not be equivariant to the inputted global orientation of the protein-ligand complex, which is not desirable for docking applications. This decision was however driven by the fact that QuickBind uses an existing protein structure instead of predicting it from scratch. For a co-folding model, abandoning reference frames and SE(3)-equivariance is therefore consistent with our findings.

Second, AF3 replaces the Evoformer with the Pairformer module. QuickBind's modified Evoformer is architecturally a middle ground between the two. Similar to the Pairformer, it operates only on single and pair representations without column-wise attention. In the case of QuickBind, our design was informed by its use of an input protein structure, which obviated the need for a multiple sequence alignment and corresponding representation. In the Pairformer, the single representation does not update the pair representation via the outer product mean (OPM) module, and the update order of the single and pair representations is swapped. In AF-Multimer [32], the OPM is moved to the beginning of the Evoformer. We found the original OPM position to be more optimal (see section SI.4), but did not try omitting it, or swapping the single and pair update order.

Third, AF3 uses larger crop sizes than AF2 and AF-Multimer. AF3 is initially trained with a crop size of 384 then finetuned in two stages with crop sizes of 640 and 768, whereas AF2 and AF-Multimer were trained with crop sizes of 256 and 384 then finetuned on crop sizes of 384 and 640, respectively. We initially trained QuickBind on 256 residue crops, then finetuned it using 512 residue crops. We found finetuning with larger crops to be important for model performance, observing consistent improvements as crop sizes increased. Given AF3's training procedure, this suggests that QuickBind would benefit from additional finetuning stages with incrementally larger crops.

Fourth, AF3 randomly chooses from contiguous, spatial, and spatial interface cropping. QuickBind's cropping strategy, binding site cropping, can be considered a compromise between AF3's two spatial cropping strategies and contiguous cropping. We tested spatial and contiguous cropping but found that binding site cropping leads to better results. Unlike QuickBind, AF3 cropping is applied to all tokens such that the model may only see parts of the ligand, while in QuickBind only the protein is cropped.

Fifth, AF3 contains a distogram head similar to the one in AF2, including a minimum distance bin of 2Å which is overly large for small molecules (C-C bonds are 1.54Å long). This suggests it primarily benefits overall complex prediction and rough positioning of ligand atoms, consistent with our observation that a distogram head does not improve QuickBind's performance.

Finally, in agreement with the fact that we found better results when not distinguishing different bond types in the ligand adjacency matrix, AF3's bond features are binary.
